# Health Impact from Air Pollution in Thailand: Current and Future Challenges

**DOI:** 10.1289/ehp.1103728

**Published:** 2011-05

**Authors:** Nuntavarn Vichit-Vadakan, Nitaya Vajanapoom

**Affiliations:** Faculty of Public Health, Thammasat University, Rangsit, Pathumthani, Thailand, E-mail: nuntavarn@tu.ac.th

Emerging from an agricultural base to more industrialization, Thailand now faces many environmental problems, particularly air pollution, resulting in adverse health consequences. The three major sources of air pollution are vehicular emissions in cities, biomass burning and transboundary haze in rural and border areas, and industrial discharges in concentrated industrialized zones.

Recent air quality data suggest that particulate matter < 10 μm in aerodynamic diameter (PM_10_) is the most important air pollutant in urban and rural areas. In cities such as Bangkok, air quality monitoring performed by the Pollution Control Department (PCD) for the past 10 years revealed that the levels of PM_10_ have exceeded both annual (50 μg/m^3^) and 24-hr (120 μg/m^3^) national standards (PCD 2010). The main source of PM_10_ in Bangkok is vehicular emissions (Chuersuwan 2008; Parsons International Ltd. 2001).

In the rural and border areas, most notably Chiangmai, agricultural burning and forest fires, including transboundary haze from Myanmar, have contributed to high levels of PM_10_, which have increased to critical levels since 2006 [250 μg/m^3^, 300 μg/m^3^, 175 μg/m^3^, and 220 μg/m^3^ in 2006, 2007, 2008, and 2009, respectively (PCD 2010)]. More importantly, many consecutive days of high PM_10_ levels resulted in increases in hospital admissions and outpatient visits (Chiangmai Provincial Public Health Office 2007).

Moreover, the Southeast Asian haze that originated in Indonesia has continually affected the health of residents of the southern provinces, particularly in 1996 and 1997, where the maximum PM_10_ levels reached as high as 314 μg/m^3^ (PCD 2010). The most severe haze episode occurred in 1997 and resulted in sharp increases in outpatient visits (26%) and hospital admissions (33% for all respiratory, 36% for pneumonia, 40% for bronchitis/chronic pulmonary obstructive disease, 12% for asthma) within a period of a few months (Health Systems Research Institute 1998).

Several studies worldwide have demonstrated that PM_10_ is associated with premature mortality and a wide range of morbidity outcomes. As part of the Public Health and Air Pollution in Asia (PAPA) multicities study, results for Bangkok showed that each 10-μg/m^3^ increase in PM_10_ is associated with a 1.25% increase in all-cause mortality, which is higher than for the three other participating cities [0.53% for Hong Kong, 0.26% for Shanghai, and 0.43% for Wuhan (Wong et al., 2008)] and higher than multicities studies conducted in Western countries (Katsouyanni et al. 2001). The higher effects in Bangkok may be related to high temperatures in Bangkok throughout the year, higher exposures to air pollution from longer periods of time spent outdoors, and less availability and use of air-conditioning.

As a developing country, Thailand strives for a continual economic growth. Consequently, expansion of petrochemical plants rose sharply, particularly, in the coastal province of Rayong, with > 73 million tons of chemicals used annually (Department of Industrial Works 2010). Although environmental management has been instituted, levels of volatile organic compounds (VOCs) continue to exceed Thailand’s standards (PCD 2010).

Epidemiological studies have been limited, with only sporadic reports of respiratory symptoms and illnesses after episodic events of accidental releases from industries. However, a recently completed large-scale population-based epidemiology study with > 26,000 subjects indicated that residents who live near the petrochemical industrial estate have higher risks in adverse pregnancy outcomes, neuropsychological symptoms, and poor performance on neuropsychological tests (Vichit-Vadakan et al. 2010). In particular, results show significant excess risk of preterm birth before < 34 weeks among mothers residing < 4 km from the industrial estate [odds ratio (OR) = 3.34; 95% confidence interval (CI), 1.18–9.47]; nonsignificant increased risks were found for all pregnancy outcomes (OR = 1.60; 95% CI, 0.87–2.93), preterm birth before 37 weeks (OR = 1.68; 95% CI, 0.85–3.30), low birth weight (OR = 1.42; 95% CI, 0.52–3.78), and small for gestational age (OR = 1.24; 95% CI, 0.31–4.90. Generally, the excess risk decreases with increased distances.

Obtaining sustainable development that balances environmental conservation and the well-being of the population remains a challenge for Thailand. In national strategies for development, policy makers often rely only on economic information fbecause of the lack of empirical data on health, social, and environmental impacts from developmental policies and projects. Fostering and strengthening epidemiological research in Thailand not only provides the necessary perspective for policy development but contributes to the larger body of knowledge in environmental health.

## Figures and Tables

**Figure:**
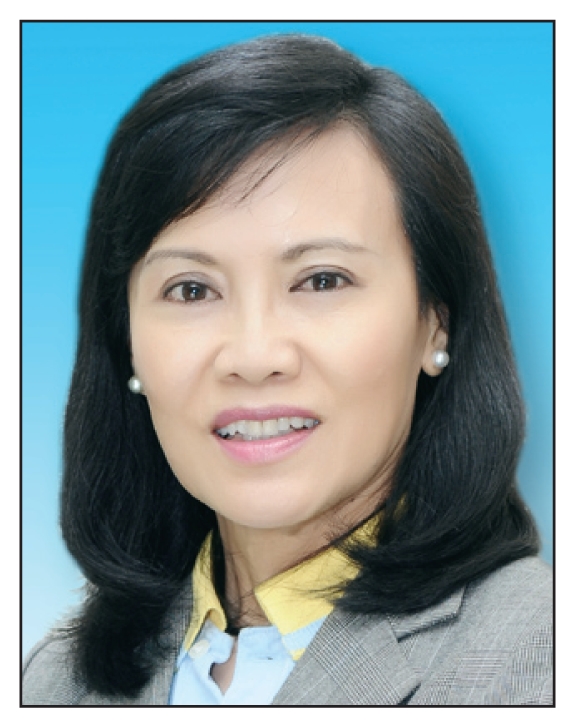
Nuntavarn Vichit-Vadakan

**Figure:**
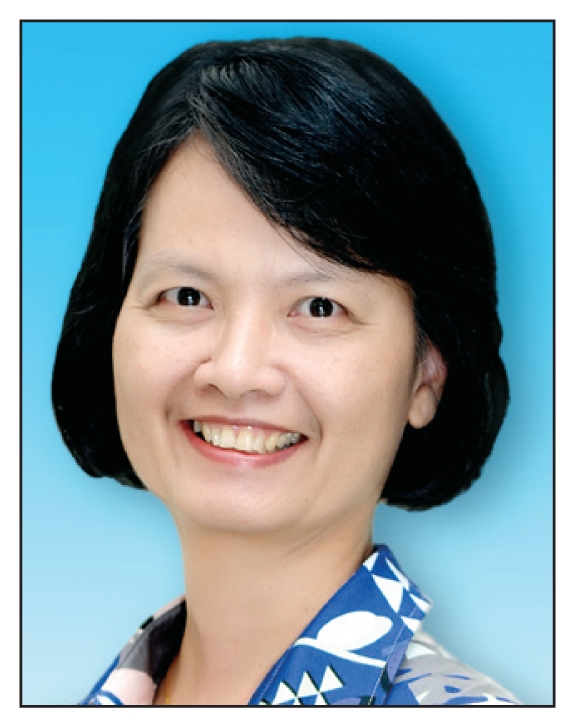
Nitaya Vajanapoom
